# Tomato and Pepper Leaf Parts Contribute Differently to the Absorption of Foliar-Applied Potassium Dihydrogen Phosphate

**DOI:** 10.3390/plants12112152

**Published:** 2023-05-29

**Authors:** Jon Niklas Henningsen, Héctor Alejandro Bahamonde, Karl Hermann Mühling, Victoria Fernández

**Affiliations:** 1 Institute of Plant Nutrition and Soil Science, Kiel University, Hermann-Rodewald-Straße 2, 24118 Kiel, Germany; khmuehling@plantnutrition.uni-kiel.de; 2 Facultad de Ciencias Agrarias y Forestales, Universidad Nacional de La Plata, Diagonal 113 N_469, La Plata 1900, Argentina; bahamondehector2019@gmail.com; 3 Systems and Natural Resources Department, School of Forest Engineering, Universidad Politécnica de Madrid, 28040 Madrid, Spain

**Keywords:** tomato, pepper, leaf surface, foliar absorption, trichomes, stomata, cuticula, phosphorus foliar spray

## Abstract

Foliar fertilisation is an application technique that is increasingly being used in agriculture and offers the possibility of providing nutrients directly to the site of highest demand. Especially for phosphorus (P), foliar application is an interesting alternative to soil fertilisation, but foliar uptake mechanisms are poorly understood. To gain a better understanding of the importance of leaf surface features for foliar P uptake, we conducted a study with tomato (*Solanum lycopersicum*) and pepper (*Capsicum annuum*) plants, which have different leaf surface traits. For this purpose, drops of 200 mM KH_2_PO_4_ without surfactant were applied onto the adaxial or abaxial leaf side or to the leaf veins and the rate of foliar P absorption was evaluated after one day. Additionally, leaf surfaces were characterised in detail by transmission electron microscopy (TEM) and scanning electron microscopy (SEM), estimating also leaf surface wettability and free energy, among other parameters. While the leaves of pepper hardly contained any trichomes, the abaxial side and the leaf veins of tomato leaves were densely covered with trichomes. The cuticle of tomato leaves was thin (approximately 50 nm), while that of pepper was thick (approximately 150–200 nm) and impregnated with lignin. Due to the fact that trichomes were most abundant in the leaf veins of tomato, dry foliar fertiliser drop residues were observed to be anchored there, and the highest P uptake occurred via tomato leaf veins, resulting in 62% increased P concentration. However, in pepper, the highest rate of P absorption was recorded after abaxial-side P treatment (+66% P). Our results provide evidence that different leaf parts contribute unequally to the absorption of foliar-applied agrochemicals, which could potentially be useful for optimising foliar spray treatments in different crops.

## 1. Introduction

Phosphorus (P) is an essential element for plants, which plays multiple structural, metabolic, physiological and signalling roles [1]. In many cropping systems worldwide, however, plant availability of the macronutrient P is limited [1,2]. This limitation is caused primarily by P sorption processes in the soil and can lead to a significant reduction in crop yield and quality due to a decrease in leaf expansion, number of leaves [3] and limited development of reproductive organs [4,5]. Furthermore, P accumulated in the soil through sorption has the potential to damage aquatic ecosystems in particular through diffuse P losses [6,7,8].

Foliar P application is an alternative form of delivering P to plants, which has been evaluated in various studies with variable P uptake and plant response success rates (e.g., [9,10,11,12,13]). This form of application enables the immediate delivery of P to the site of demand and can thus not only potentially improve P use efficiency but also reduce losses. Recent studies, however, have also found that plant functionality of P-deficient maize (*Zea mays* L.) and barley (*Hordeum vulgare*) plants, especially with regard to photosynthetic processes, can be temporarily improved but not fully restored after P foliar application (e.g., [11,12]). For other nutrients, such as magnesium (Mg), which is required by plants in similar amounts to P, however, the restoration of plant functionality may be possible, as reported in some studies [14]. Apart from some hypotheses, there is no clear explanation for the low effectiveness of foliar P application despite the potential high uptake rates recorded in some investigations (e.g., [10]).

The mechanisms of foliar-applied agrochemicals have been evaluated for more than 70 years, but they are still not well characterised owning to the complex scenario and the major variability among plant surfaces from different organs, stages of development, species/varieties or growing conditions, among other factors [15]. It has been reported that especially factors such as physiological, environmental or foliar-spray-formulation-related ones influence the efficacy of foliar fertilisers [15]. Furthermore, absorption may take place via different epidermal structures, such as stomata or trichomes, in addition to the cuticle and other potential surface structures [16]. Recent studies actually point towards the differential contribution of potential uptake pathways among leaves from different species [16]. For example, using synchrotron-based X-ray fluorescence microscopy for tracing zinc (Zn) foliar uptake, the main foliar-applied Zn absorption pathway of sunflower (*Helianthus annuus*) leaves was found to be glandular trichomes [17], while, in tomato leaves, non-glandular trichomes did not seem to play a role in Zn absorption [18].

As mentioned above, stomata are one of the main epidermal structures associated with foliar fertiliser absorption. Their contribution to the foliar uptake process and potential absorption of pure water solutions has been controversial for many years [19,20,21,22,23]. While Schönherr and Bukovac [20] postulated that surfactants with a surface tension of 30 mN m^−1^ or below should be added to achieve stomatal infiltration of solutions, Eichert and co-workers [21,22,23] provided evidence for the uptake of different ions and molecules when leaf stomata are open using various plant species and experimental approaches. Depending on their size, dissolved substances can diffuse through a liquid film on the outside of the guard cells so that, for example, dissolved nutrients can enter the inside of the leaf from the leaf surface [22,23].

Aside from stomata and trichomes, the cuticle also has an important function in the transport of foliar-applied nutrients. Fertiliser spray drops will interact differently with plant surfaces according to their chemical and structural nature, particularly of the cuticle, resulting in variable degrees of wettability and drop adhesion or repellence [16,24]. Most aerial plant surfaces are covered and thereby protected by the cuticle. While the cuticle has been considered a lipid layer independent from the underlying cell wall for more than one century, recent studies highlight its cell wall nature [25,26,27]. The cuticle consists to a large extent of cutin (a polyester of C16 and/or C18 hydroxy fatty acids) but also of cell wall material (i.e., polysaccharides, pectin and hemicellulose) and intra- and epi-cuticular waxes [28,29]. Particularly, apolar waxes can significantly impair the transport of polar liquids through the plant surface [14]. It has been hypothesised that the uptake of dissolved nutrients occurs in association with polar functional groups (e.g., polysaccharides) [15]. When the cuticle hydrates, water may interact with these groups, leading to cuticle swelling and facilitating the transport of solutes across it [25,30].

The main objective of this study was to evaluate the uptake potential of different leaf lamina zones using tomato and pepper as model plant species and a pure water solution (i.e., without surfactant) with potassium (K)-dihydrogen phosphate (KH_2_PO_4_) as a foliar P fertiliser. The hypotheses to be tested were: (I) pepper and tomato leaves can absorb KH_2_PO_4_ supplied as a water solution with no surfactant; (II) different P foliar absorption rates will be recorded for tomato and pepper depending on the treated leaf lamina area; (III) the performance of water drops deposited on the leaves of tomato and pepper may provide hints about their potential for absorbing foliar-applied agrochemicals; and, finally, (IV) the differences in leaf surface topography and inner structure between tomato and pepper influence the deposition and potential absorption of foliar-applied KH_2_PO_4_.

## 2. Results

### 2.1. Leaf Surface Features

**Figure 1:**
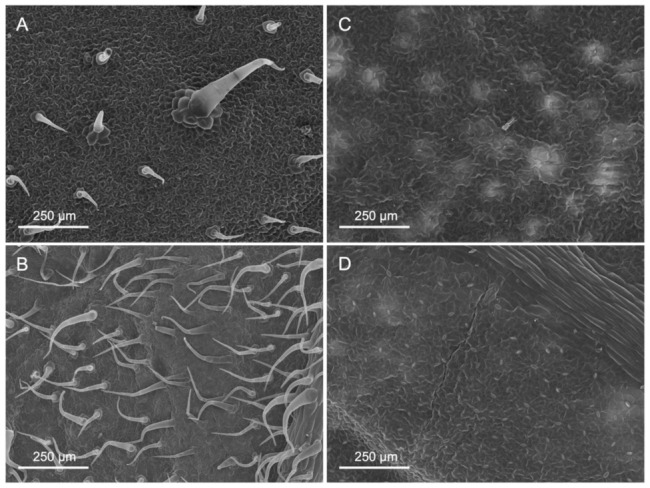
Scanning electron micrographs of untreated adaxial (**A**,**C**) and abaxial (**B**,**D**) surfaces of tomato (**A**,**B**) and pepper (**C**,**D**) leaves.

**Figure 2:**
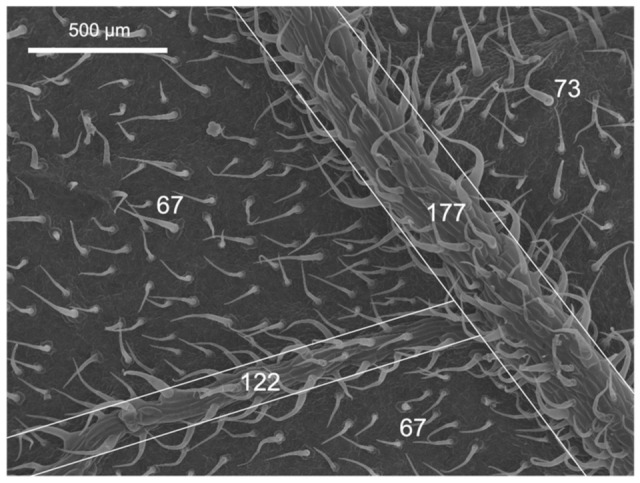
Scanning electron micrograph of the abaxial leaf surface of tomato. The white numbers indicate the N° of trichomes mm^−2^ in the areas marked with white borders.

**Figure 3:**
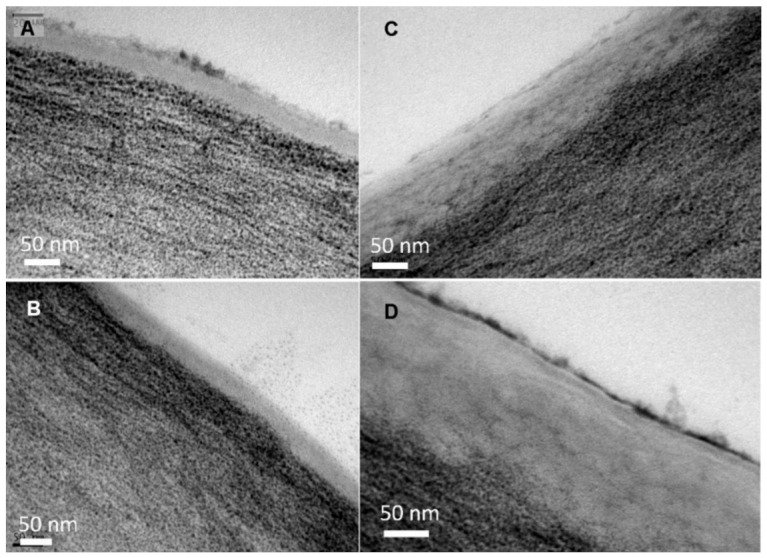
Transmission electron micrographs of the adaxial (**A**,**C**) and abaxial (**B**,**D**) lamina cuticle and cell wall of tomato (**A**,**B**) and pepper (**C**,**D**) leaves.

The main surface features of the upper (adaxial) and lower (abaxial) leaf side of the pepper and tomato leaves analysed are shown in Table 1 and Figure 1, Figure 2 and Figure 3.

Tomato leaves have non-glandular trichomes with high densities in the abaxial leaf lamina (83.7 mm^−2^), also including the central vein (Table 1 and Figure 1). On the adaxial side, on the other hand, with 16.5 trichomes mm^−2^, there are considerably less. Furthermore, the trichome density on the abaxial side of tomato leaves is markedly higher on the veins than on the leaf lamina (Figure 2). Pepper leaf surfaces are glabrous in the adaxial lamina and occasionally have some glandular trichomes on the abaxial side (2.7 mm^−2^). Both species are amphistomatous, with higher densities occurring on the abaxial side of tomato and pepper leaves. The adaxial surface of tomato leaves only has a few stomata, while approximately 52 mm^−2^ were recorded for pepper leaves (Table 1, Figure 1). Apart from stomata and trichomes, no structures potentially influencing physico-chemical properties (e.g., epicuticular waxes/crystalloids) were found on the leaf surfaces of tomato and pepper.

**Figure 4:**
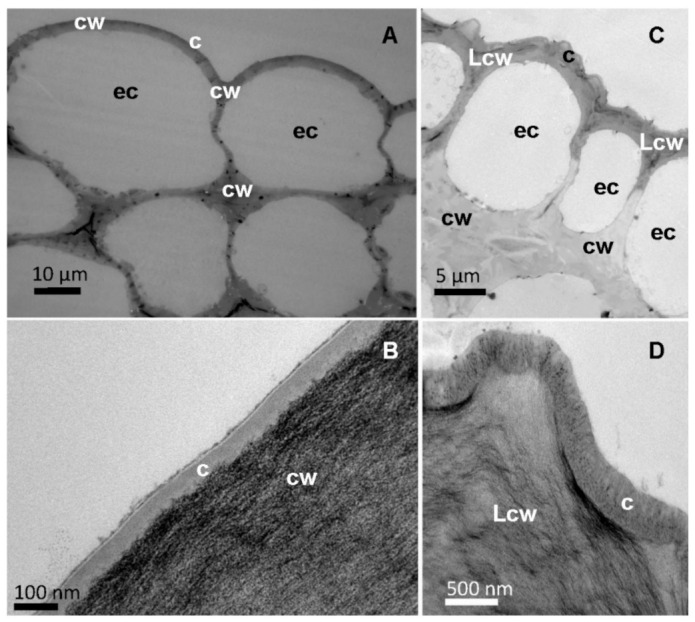
Cross-sections of the central vein of tomato (**A**,**B**) and pepper (**C**,**D**) leaves observed by TEM at different magnifications, (**B**) and (**D**) focussing on the epidermal cell wall. Letters indicate epidermal cells (ec). Cuticle (c), cell wall (cw) and lignified cell wall (Lcw).

When observing the structure of tomato and pepper leaf cross-sections by TEM (Figure 3 and Figure 4), with focus on the epidermal cell wall, a thin cuticle of approximately 50 nm was found in the adaxial and abaxial leaf lamina of tomato. By contrast, pepper leaves have a thicker cuticle, which was approximately 150 and 200 nm for the adaxial and abaxial sides, respectively.

When observing the surface of the major leaf veins, a cuticle of similar thickness to that covering the leaf lamina could also be identified as the external part of the epidermal cell wall (Figure 4). The tomato vein surface is smooth compared to the rough surface of pepper veins due to the occurrence of cuticular folds (Figure 4A vs. Figure 4C). In addition, some epidermal cell wall areas of pepper veins appear dark (Figure 4C), and this can be associated with the deposition of lignin, which can be identified as a dark material deposited in the cuticle area (Figure 4D).

### 2.2. Contact Angles and Derived Surface Free Energy Parameters

The contact angles of the different leaf surfaces of both species, with three liquids having variable polar/apolar components, are shown in Table 2. In tomato plants, the adaxial lamina was found to be more wettable for water and diiodomethane. Moreover, the abaxial lamina and vein showed high contact angles with water and can hence be considered poorly wettable. For glycerol, the abaxial vein was more unwettable than the leaf laminas (adaxial and abaxial). In pepper plants, all the leaf parts are wettable for water, glycerol and diiodomethane, although the abaxial vein had contact angles slightly higher than 90° (Table 2).

Advancing water contact angles were higher for the abaxial lamina of tomato leaves, while, in pepper, no differences were detected between leaf sides (Table 3). In the case of receding water angles, again, significant differences between leaf sides were recorded for tomato while pepper showed similar values. The contact angle hysteresis (i.e., difference between advancing and receding angles) was higher for abaxial lamina in tomato leaves, indicating major adhesion of water drops to this leaf part compared to the adaxial leaf side (Table 3). In pepper leaves, the contact angle hysteresis of water drops was similar for adaxial and abaxial lamina surfaces.

The surface free energy (*γ_s_*) and its components (*γ^LW^* and *γ^AB^*) were calculated for different leaf parts of the two species analysed. In tomato, the abaxial lamina and vein had low *γ_s_*, *γ^LW^* and *γ^AB^* values, which can be associated with the low wettability of such surfaces by polar water and glycerol drops (Table 4). This pattern was coherent with the lower polarity and solubility parameter calculated for these leaf parts. On the other hand, in pepper plants, adaxial and abaxial lamina surfaces showed lower surface-free-energy-related values (excepting the apolar component, *γ^LW^*) compared to the abaxial vein, reflecting the interactions of these foliar surfaces with the different liquids evaluated.

### 2.3. Foliar KH_2_PO_4_ Deposition and Absorption

**Figure 5:**
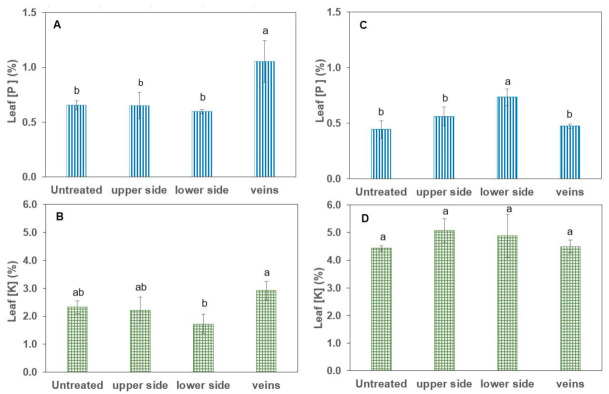
Tissue P and K concentrations of tomato (**A**,**B**) and pepper (**C**,**D**) plants one day after foliar application of 200 mM KH_2_PO_4_ with no surfactant. Values are mean ± SD (*n* = 4). For the same species and applied element, different letters indicate significant differences between treated leaf parts according to Tukey’s HDS test (*p* ≤ 0.05).

A pure distilled water solution (i.e., with no surfactant) of 200 mM KH_2_PO_4_ was supplied to different leaf areas by applying approximately 3 µL drops at a density of 3.4 cm^−2^ (tomato) to 3.7 cm^−2^ (pepper) for the adaxial and abaxial leaf lamina as well as to the leaf veins of tomato and pepper (Figure S1). The P and K leaf concentrations were evaluated one day after treatment, as shown in Figure 5.

For tomato leaves, a significant absorption of P was only detected in association with vein drop deposition of the foliar treatment (+62% P). However, pepper leaves only significantly absorbed P when treatment solution drops were deposited onto the abaxial (lower) leaf side (+66% P), while no significant evidence of uptake was gained for K in comparison to untreated control plants of pepper and tomato.

**Figure 6:**
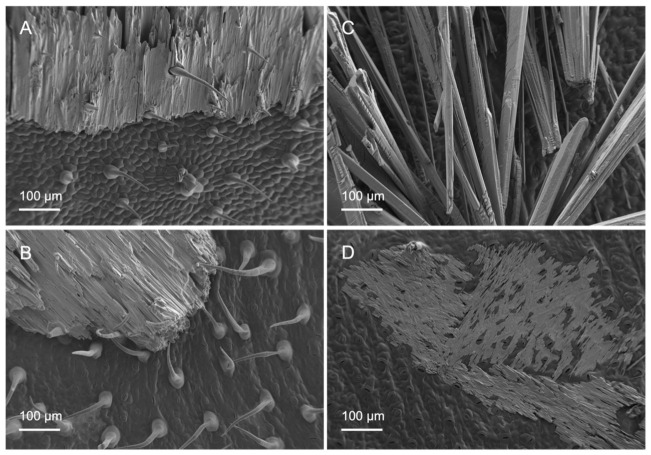
Scanning electron micrographs of adaxial (**A**,**C**) and abaxial (**B**,**D**) surfaces of tomato (**A**,**B**) and pepper (**C**,**D**) leaves with different forms of salt deposition after foliar application of 200 mM KH_2_PO_4_ with no surfactant.

The appearance of KH_2_PO_4_ deposits after foliar treatment solution drop drying was assessed by SEM, as shown in Figure 6. Dry salt deposits acquired different structures, with different drop crystallisation patterns being observed for tomato versus pepper leaf surfaces. The drop deposits occurring on tomato leaf surfaces were thick and extensive and were usually firmly attached to the trichomes, which prevented them from falling off the leaves (Figure 6A,B). On the other hand, on pepper leaves, salt crystals often were not held by the surfaces and fell off when the leaf was shaken. Furthermore, there were deposits whose salt crystals were long and finely structured and rather diffusely arranged (Figure 6C), which made them more likely to slip off the leaf surface. Another type of dry deposits observed of pepper leaves corresponded to a flat small-area distribution of salt crystals, with stomata still remaining partially visible (Figure 6D).

## 3. Discussion

Foliar P fertilisation has the potential to provide plants with P at the site of highest nutrient demand. However, the factors influencing P uptake into the plant are not fully understood yet [16]. Therefore, this study aimed at determining the uptake potential of different leaf zones depending on their morphological and chemical properties using pepper and tomato as model plants.

According to Bergmann [31], fully developed, well-nourished leaves of tomato and pepper plants should contain a P concentration of 0.4 to 0.65% and 0.3 to 0.6%, respectively. Hence, the untreated control plants of tomato (0.65% P), as well as pepper (0.44% P), can be considered as sufficiently P-supplied (Figure 5A,C). Nevertheless, a significant amount of P was taken up via the veins in tomato and through the abaxial leaf side in pepper as a result of the pure water KH_2_PO_4_ foliar application (Figure 5A,C). No P uptake was observed via the remaining leaf parts, and also no significant K uptake took place over any of the leaf parts compared to the untreated control variants (Figure 5). The lack of evidence for foliar K absorption, despite the rather high concentration of 200 mM KH_2_PO_4_ in the foliar fertiliser solution, was recently confirmed by Bahamonde et al. [32] and was attributed by the authors to potential factors such as a major physiological role of K for stomatal aperture, high plant mobility or interactions with membrane transporters. The selective uptake of P via specific leaf parts is most probably related to the different physicochemical properties of these leaf areas, as discussed in more detail below.

The leaves of tomato and pepper plants differed not only between them but also within the two species in terms of the different leaf parts. While the number of stomata on the abaxial leaf side was similar in both species, pepper formed more stomata on the adaxial side than tomato (Table 1). However, trichomes were formed on both sides of tomato leaves in significantly greater numbers than in pepper, where hardly any trichomes occurred (Figure 1, Table 1). The number of trichomes significantly influences the micro-scale roughness of plant surfaces [16,33,34,35] and may thus be linked to the low wettability of tomato leaves (Table 2). Furthermore, a decrease in wettability with increasing trichome density can be observed among the different leaf parts of tomato (adaxial lamina > abaxial lamina > veins), which supports the previous assumption (Figure 1, Table 2). The mostly glabrous pepper leaf surfaces had relatively low contact angles and were thus wettable (Table 2) [16]. Tomato leaf sections with low wettability/high trichome density nevertheless showed the highest rate of P absorption, as derived from the high contact angle hysteresis determined (Table 3). However, although trichomes have previously been linked to water and solute uptake (e.g., [17,36]), Li et al. [18] demonstrated that no foliar-applied Zn was taken up via tomato trichomes, making it questionable that trichomes were the main path of P uptake in our case. For pepper, on the other hand, it is plausible that stomata on the abaxial leaf side were associated with the greater P uptake recorded as all other physicochemical features did not differ between either leaf side of pepper plants.

Even though trichomes might not be significantly involved in nutrient uptake in tomato leaves, they nevertheless fulfilled another role. Probably, after the initially poor water drop, tomato vein and abaxial leaf surface interactions, the liquid of the treatment drops penetrated the trichome network, as derived from the appearance of the salt deposits, as observed by SEM (Figure 6A,B). It became evident that, after drying of the applied drops, the trichomes stuck out of the formed salt crystals and seemed to anchor them to the surface (Figure 6A,B). This means that there was permanent contact between the nutrient and the leaf surface and thus the possibility of uptake in the event of rewetting of the foliar fertiliser as a result of high relative humidity. Since most trichomes occurred on the leaf veins (Figure 2), foliar fertiliser residues were probably best anchored here. Furthermore, Arsic et al. [37] found in barley plants that foliar P uptake mainly took place via fibre cells and the subsequent translocation via the underlying bundle sheath extensions. Moreover, a tiny reticulate cuticle was observed to cover the tomato leaf surfaces, which enabled the absorption of P by the leaves when drops were deposited onto the veins (Figure 3A,B). By contrast, the smoother pepper leaf surface covered with amorphous waxes, probably in a more uniform manner, led to a different KH_2_PO_4_ crystallisation structure and enabled deposits to fall off the leaves if they were shaken (Figure 6C,D). Despite being apparently more wettable but rougher, the pepper vein epidermal cell wall was lignifying/lignified and may be more apolar and a cell wall made out of cellulose, pectin and hemicellulose, which are polymers prone to polar and H-bonding interactions (Figure 4C,D, Table 2) [25,38]. The secretion of lignin to the epidermal cell wall of pepper veins may limit the transport of water and solutes compared to a primary cell wall and a cuticle area, as observed in pavement epidermal cells.

The total surface free energy of pepper leaves is within the range reported for smooth leaf surfaces, such as pepper fruit or *Ficus elastica* leaf [24]. However, the total leaf surface values of veins of pepper and adaxial leaf side of tomato are higher, which is accompanied by a higher polarity percentage in these materials. This may be related to the occurrence of polar areas in such surfaces, but this must be studied in greater detail in future investigations. The solubility parameter value of adaxial and abaxial pepper leaves corresponds to that estimated for waxes [38]. The abaxial leaf lamina and vein of tomato were unwettable by water, which is associated with surface chemical composition and the roughness chiefly conferred by trichomes. This leads to solubility parameter values slightly below those theoretically estimated for waxes (i.e., 15–16 MJ^1/2^ m^−3/2^) (Table 4).

In conclusion, we evaluated the physico-chemical properties of different leaf lamina areas of pepper and tomato plants that were rather glabrous or had many trichomes, respectively. The adaxial side of tomato and both sides of pepper leaves had water contact angles of approximately 84°, while the abaxial side and veins of tomato leaves were unwettable by water. Despite the apparently higher contact angles of these tomato lamina areas with water, they had high water contact angle hysteresis values, which suggests a role of trichomes in the retention of water drops. After applying drops of 200 mM KH_2_PO_4_ supplied in the absence of surfactant, we only gained evidence of foliar P uptake after depositing drops on the veins of tomato leaves and onto the lower side of pepper leaves. No significant evidence of K uptake was gained in comparison to untreated control plants. The vein cuticle of tomato leaves was observed to be thin and reticulate compared to the lignified and rougher surface of pepper veins. Drop KH_2_PO_4_ deposits after drying led to different patterns of crystallisation depending on the treated pepper and tomato leaf areas, which were differently retained by the surfaces. It is concluded that the surface features and physicochemical characteristics of leaves and different leaf lamina zones may be preferential sites for the absorption of foliar-applied agrochemicals and that it is possible that treatments supplied to the foliage in the absence of surfactants are taken up depending on leaf surface wettability, structure and chemical composition.

## 4. Materials and Methods

### 4.1. Plant Material

Tomato (*Solanum lycopersicum*, cv. Bolar) and pepper (*Capsicum annuum*, cv. Lamuyo) plants were germinated in a nursery (Semilleros el Mirador S.L., El Mirador, Spain) and thereafter raised under controlled conditions and with sufficient nutrient and water supply in a greenhouse in Madrid (40°27′05.3″ N, 3°43′22.5″ W, Spain) from May to June. The temperature was controlled by automatic air exchange and ranged from 20 °C at night to a maximum of 30 °C during the day. Light and relative humidity corresponded to the environmental conditions. Thus, the plants were exposed to a day length of 14.5–15 h and a relative humidity of 35–70% during the day and up to 100% at night. The plants were arranged in a fully randomised design.

After ten weeks of growth at a plant height of 50–70 cm, a single P (KH_2_PO_4_) foliar drop application was conducted on the tomato and pepper plants (described in Section 4.4). Shortly before applying the P drops, several leaf characteristics were investigated using the methods described below, and, 24 h after application, homogeneous, intact and fully developed leaves were sampled for nutrient analysis in four replicates per variant.

### 4.2. Electron Microscopy

For a precise characterisation of the surface, leaf segments were analysed either intact (FESEM) or after prior fixation, dehydration and critical point drying (SEM). Furthermore, TEM images were taken to investigate the cell structure and properties of the cuticle and cell wall.

For the purpose of SEM analysis, segments of about 10 mm^2^ were first cut from intact lamina tissue using a scalpel before being immersed directly in phosphate buffer (pH 7.2). After 4 h of storage in phosphate buffer, a dehydration series with ethanol (30, 50, 70, 80, 90, 100%) (Merck, Darmstadt, Germany) and distilled water was initiated, followed by critical point drying (Leica EM CPD300, Leica Microsystems, Wetzlar, Germany). Finally, the samples were sputtered with gold (Au) and observed in SEM (Jeol JSM6400, Jeol Ltd., Akishima, Japan) at the National Electron Microscopy Centre (CNME, UCM, Madrid, Spain). In order to further characterise the deposits on the leaf surface caused by the application of KH_2_PO_4_, untreated 10 mm^2^ leaf segments were examined by FESEM at Miguel Hernandez University of Elche (UMH, Elche, Spain).

For TEM, small pieces of 4 mm^2^ were cut from the leaf lamina, margin and veins and were first stored in phosphate buffer for 6 h before being transferred to a 1:1 solution of osmium tetroxide (2%, TAAB Laboratories, Aldermaston, UK) and potassium ferrocyanide (3%, Sigma- Aldrich, St. Louis, MO, USA) for 1.5 h. Subsequently, the samples were dehydrated in an acetone series (30, 50, 70, 80, 90, 95, 100%) (Merck, Darmstadt, Germany) and thereafter immersed in an acetone–Spurr’s resin (TAAB Laboratories, Aldermaston, UK) mixture (3:1 for 2 h, 1:1 for 2 h, 1:3 for 3 h, pure resin for 12 h) of increasing concentration. With pure resin, the samples were placed in a mould and kept in the oven at 70 °C until complete polymerisation of the Spurr’s resin. As a final step, ultra-thin sections were cut (Leica Ultracut E, Leica Microsystems, Wetzlar, Germany) and the leaf cross-sections were then observed by TEM (Jeol JEM 1400plus, Jeol Ltd., Akishima, Japan) in the CNME (UCM, Madrid, Spain).

### 4.3. Contact Angles and Derived Parameters

Equilibrium and dynamic contact angles were determined for the adaxial and abaxial leaf sides as well as for the veins of tomato and pepper plants to obtain detailed information on leaf wettability and other physico-chemical surface properties.

Therefore, a 1 mL syringe with a 0.5 mm diameter needle was used to deposit 15 drops (*n* = 15) of approximately 2 µL of de-ionised water, glycerol (99% purity, Sigma-Aldrich, St. Louis, MO, USA) and diiodomethane (99% purity, Sigma-Aldrich, St. Louis, MO, USA) on the respective plant part, which was fixed to a microscope slide with double-sided adhesive tape. Contact angles were automatically calculated with a drop shape analysis system (DSA 100, Krüss, Hamburg, Germany) using the tangent equation and resulting surface properties were characterised with the 3-liquids method [39], taking into account the previously mentioned liquids and their differences in the degree of dispersive and non-dispersive components.

Advancing (*θ_adv_*) and receding (*θ_rec_*) contact angles of water were measured under a similar setup as the equilibrium contact angles. For the determination of *θ_adv_*, a drop was placed on the plant surface and remained in contact with the needle of the syringe. Then, the volume of the drop was continuously increased until the contact line between drop and leaf surface was displaced (about 4–5 µL for pepper, about 6 µL for tomato), and the contact angle was measured just prior to this shifting. *θ_rec_* was determined when the volume of the drop was decreased but the contact with the needle and the leaf surface still remained (about 1 µL for pepper and tomato). The measurements were repeated 12 times (*n* = 12) on the adaxial and abaxial sides of pepper and tomato leaves.

### 4.4. Foliar KH_2_PO_4_ Treatment

For assessing the potential uptake of a pure (i.e., without surfactant) P-foliar fertiliser by different leaf areas, drops of approximately 3 µL of 200 mM KH_2_PO_4_ (Sigma-Aldrich-Merck, Darmstadt, Germany) were deposited in zones of the adaxial and abaxial leaf lamina, and also onto leaf veins. In all cases, the entire zone to be treated was covered with drops of KH_2_PO_4_, so, for tomato leaves, a total of approximately 90 drops were placed onto the adaxial lamina, 140 onto the abaxial lamina and 135 onto the veins. For the pepper leaves, 110 drops were applied on to the adaxial lamina, 95 on the abaxial lamina and 150 on the veins.

### 4.5. Leaf P and K Determination

One day after the foliar P drop application, ten leaves per replicate were sampled for analysis of P and K concentrations in the plant tissue and were first cleaned from adhering nutrient salts in a three-step washing procedure with tab and distilled water [32]. Subsequently, the leaves were oven-dried at 65 °C until they were constant in weight and then ground prior to mineral element determination by inductively coupled plasma optical emission spectroscopy (ICP-OES, Optima 3000, PerkinElmer, Waltham, MA, USA), following the UNE-EN ISO/IEC 17025 standards for calibration and testing laboratories (CEBAS-CSIC Analysis Service, Murcia, Spain) as previously described by Bahamonde et al. [32].

### 4.6. Statistical Analysis

Firstly, exploratory analyses of the data were carried out to verify compliance with the assumptions of homoscedasticity and normality of the data for each situation evaluated. To verify the homoscedasticity of the variances of the data, a Bartlett’s test was performed. In case the variances were not homogeneous, data transformations were performed. Kolmogorov–Smirnov (KS) test was used to check the normality of the data.

To evaluate potential differences in stomatal and trichome density between leaf sides, a Student’s *t*-test was performed.

Differences in leaf surface features (contact angles) and nutrients (K and P) concentrations were analysed by performing one-way ANOVA, with leaf parts as main factor. Tukey HDS and Student’s *t*-tests were carried out for estimating differences between factors when F-values were significant (*p* ≤ 0.05). The analyses were carried out separately for each species.

Statistical analyses were performed using R (version 4.2.1, R Foundation for Statistical Computing, Vienna, Austria).

## Figures and Tables

**Table 1:** Stomatal and trichome densities of tomato and pepper leaves.

Species	Leaf Side	*Stomatal Density* (Nº mm^−2^)	*Trichome Density* (Nº mm^−2^)
Tomato	Adaxial	1.8 ± 0.6 a	16.5 ± 0.8 a
	Abaxial	189.0 ± 5.1 b	83.7 ± 2.4 b
Pepper	Adaxial	51.8 ± 1.7 a	0.0 ± 0.0 a
	Abaxial	169.4 ± 4.1 b	2.7 ± 0.1 b

Values are mean ± standard error (SE) (*n* = 4). For the same species, lowercase letters indicate significant differences between leaf parts according to Student’s *t*-test (*p* ≤ 0.05).

**Table 2:** Contact angles of water (***θ_w_***), glycerol (***θ_g_***) and diodomethane (***θ****_d_*) with adaxial and abaxial leaf surfaces of tomato and pepper plants.

Species	Leaf Part	*θ_w_* (°)	*θ_g_* (°)	*θ_d_* (°)
Tomato	Adaxial lamina	84.2 ± 0.4 a	96.7 ± 0.3 a	57.7 ± 0.3 a
	Abaxial lamina	118.0 ± 0.4 b	108.6 ± 0.4 a	74.3 ± 0.4 b
	Abaxial vein	128.8 ± 0.5 b	121.7 ± 0.6 b	77.7 ± 0.6 b
Pepper	Adaxial lamina	83.5 ± 0.4 a	80.7 ± 0.4 a	65.0 ± 0.3 a
	Abaxial lamina	84.6 ± 0.4 a	84.7 ± 0.3 a	61.8 ± 0.3 a
	Abaxial vein	74.0 ± 0.2 a	91.1 ± 0.2 b	64.7 ± 0.3 a

Values are mean ± SE (*n* = 20). For the same species, different letters indicate significant differences between leaf parts according to Tukey’s HDS test (*p* ≤ 0.05).

**Table 3:** Advancing (*θ_adv_*), receding (*θ_rec_*) and contact angle hysteresis (Δ*θ**_w_***) of water drops with adaxial and abaxial leaf lamina surfaces of tomato and pepper plants.

Species	Leaf Side	*θ_adv_* (°)	*θ_rec_* (°)	Δ*θ_w_* (°)
Tomato	Adaxial	94.4 ± 0.6 a	28.3 ± 0.2 a	66.1 ± 0.4 a
	Abaxial	136.4 ± 0.7 b	46.6 ± 0.6 b	89.8 ± 0.7 b
Pepper	Adaxial	85.7 ± 0.5 a	29.1 ± 0.2 a	56.6 ± 0.3 a
	Abaxial	85.6 ± 0.6 a	27.7 ± 0.3 a	58.0 ± 0.5 a

Values are mean ± SE (*n* = 15). For the same species, different letters indicate significant differences between leaf parts according to Tukey’s HDS test (*p* ≤ 0.05).

**Table 4:** Lifshitz van der Waals component (*γ^LW^*), acid–base component (*γ^AB^*), total surface free energy (*γ_s_*), polarity (*γ^AB^ γ*^−1^) and solubility parameter (*δ*) of adaxial and abaxial leaf surfaces of tomato and pepper plants.

Species	Leaf Part	*γ^LW^* (MJ m^−2^)	*γ^AB^* (MJ m^−2^)	*γ_s_* (MJ m^−2^)	*Polarity* (%)	*δ* (MJ^1/2^ m^−3/2^)
Tomato	Adaxial lamina	29.9	15.2	45.1	33.6	21.6
	Abaxial lamina	20.5	0.5	21.0	2.2	12.1
	Abaxial Vein	18.7	0.4	19.1	2.0	11.33
Pepper	Adaxial lamina	25.7	1.1	26.8	4.1	14.6
	Abaxial lamina	27.5	2.2	29.7	7.4	15.8
	Abaxial Vein	25.9	14.8	40.7	36.4	20.0

## Data Availability

The data presented in this study are available on request from the corresponding authors.

## Supplementary Materials

The following supporting information can be downloaded at: https://www.mdpi.com/article/10.3390/plants12112152/s1, Figure S1: Example how P fertilizer drops were applied onto the foliage plants.
