# Supplementary material for: Tomato and Pepper Leaf Parts Contribute Differently to the Absorption of Foliar-Applied Potassium Dihydrogen Phosphate
Source: Plants (Basel). 2023 May 29;12(11):2152. doi: 10.3390/plants12112152 (PMC10255901; doi:10.3390/plants12112152)
Supplement: Supplementary file 1 [file plants-12-02152-s001.zip › plants-2426101-supplementary.pdf]

Supplemental Material

Title: Illustration of the drop application.

Authors: Jon Niklas Henningsen, Héctor Alejandro Bahamonde, Karl Hermann Mühling and Victoria Fernández

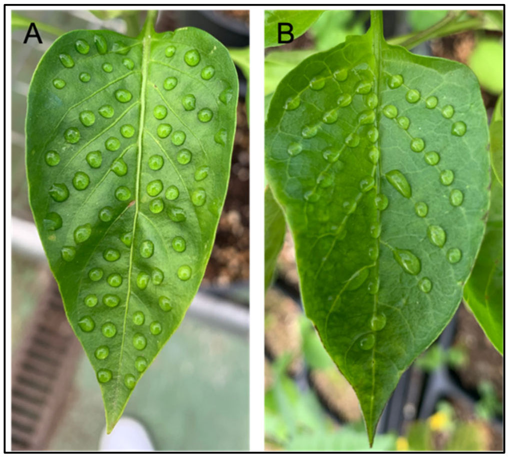

Figure S1. Drops of 200 mM KH_2PO_4 deposited on the adaxial leaf lamina (**A**) and the veins (**B**) of pepper leaves.
